# Swedish Intensive Care Physicians' Attitudes Towards Withholding or Withdrawing Life‐Sustaining Treatment in Critically Ill Children

**DOI:** 10.1111/aas.70256

**Published:** 2026-05-15

**Authors:** Maria Ahlerup, Johan Malmgren, Albert Gyllencreutz Castellheim, Johan Holmén, Ola Ingemansson, Lars Sandman, Helena Odenstedt Hergès, Linda Block

**Affiliations:** ^1^ Department of Anaesthesiology and Intensive Care Institution of Clinical Sciences, Sahlgrenska Academy, University of Gothenburg Gothenburg Sweden; ^2^ Department of Pediatric Anaesthesia/Surgical Operations/Intensive Care Queen Silvia Children's Hospital, Sahlgrenska University Hospital, Region Västra Götaland Gothenburg Sweden; ^3^ Department of Anaesthesia and Intensive Care Region Västra Götaland, Sahlgrenska University Hospital Gothenburg Sweden; ^4^ Department of Health, Medicine and Caring Sciences The National Centre for Priorities in Health, Linköping University Linköping Sweden

## Abstract

**Background:**

Decisions to withhold or withdraw life‐sustaining treatments for critically ill children are challenging. This study aims to identify Swedish intensivists' attitudes, values and experiences towards the decision‐making process concerning withholding or withdrawal of life‐sustaining treatment in critically ill children.

**Methods:**

The nine intensive care units in Sweden that treated more than 10 children for more than 48 h during 2023 were approached. A web‐based questionnaire on attitudes and experiences on end‐of‐life decision‐making in critically ill children was distributed to the 360 physicians employed at the above‐mentioned intensive care units. The results were analysed descriptively.

**Results:**

Fifty‐five answers were retrieved. The participating physicians were experienced, 93% having personal experience with end‐of‐life decision‐making in children. The main findings were that the child's wishes were considered important or very important by 93% of respondents. The guardians' wishes were considered important or very important by 82% of respondents. Seventy‐three percent of physicians considered it ethically acceptable to withdraw life‐sustaining treatment against the patients' and guardians' wishes. To continue life‐sustaining treatment without the patient's and guardian's consent was considered ethically acceptable by 26% of participants.

**Conclusions:**

This study demonstrated that Swedish intensivists share common attitudes and values regarding most issues related to end‐of‐life decisions in critically ill children. However, there is variability in attitudes concerning patients' and guardians' wishes. Further studies concerning end‐of‐life decisions in critically ill children within a Nordic context are warranted.

**Editorial Comment:**

This survey study presents a sampling of views on pediatric intensive care withholding or withdrawing of treatment and from specialist physicians in one Nordic country.

## 1 Introduction

In all medical treatment, continuous evaluation is necessary to ensure that the treatment is in the patient's best interest. This is even more evident in intensive care, where the quality of life during the actual treatment is very low. Often, the patient cannot even be fully conscious during the treatment. Even though symptomatic treatment for pain and anxiety is provided, it does not leave much space for experiences that enhance the quality of life. If the prognosis offers a good chance of recovery and discharge from the hospital with a favourable outcome, the opportunity of doing good outweighs the risk of harm. However, when the patient is in an end‐of‐life situation, care must be taken to avoid disproportionate treatment that will not benefit the patient [1, 2, 3].

Each year, 60–80 children under the age of 16 years die in Swedish intensive care units (ICUs) [4]. This corresponds to one‐fifth of the total deaths in this age group [5]. These data refer to ICUs outside of neonatal intensive care, as these patients are not within the scope of this study.

Health care in Sweden is regulated by The Swedish Medical Services Act [6]. It states the patient's right to receive information about their health condition and to receive treatment deemed indicated by the responsible physician. The patient also has the right to deny treatment if they are considered autonomous. The patient, however, does not have the right to demand treatment that is not medically indicated.

Identifying that the patient is beyond recovery and making the necessary decisions to withhold or withdraw life‐sustaining treatment (LST) is a challenging and complex process.

Studies of Swedish intensivists' attitudes towards end‐of‐life decision‐making in an adult setting have demonstrated that extensive case information and consensus among the patient, family, staff and other physicians are essential [7].

Earlier studies in a European adult ICU context have shown that perceptions of inappropriate care are common among both physicians and nurses [8]. The perception of disproportionate care can be defined as the intensity of care being inappropriate in relation to the patient's prognosis. It has been demonstrated that identifying disproportionate care correlates with the ethical decision‐making climate within the department [9].

The attitudes of paediatric intensive care professionals have been studied in a European context, with a slight variability between regions [10]. End‐of‐life decision‐making in a paediatric intensive care setting has not previously been studied in Sweden.

The primary aim of this study was to identify attitudes, values and experiences towards the decision‐making process to withhold or withdraw LST in critically ill children among Swedish intensivists. Furthermore, the aim was to determine whether these issues are perceived differently among physicians from designated paediatric intensive care units (PICUs) compared with those working in mixed ICUs.

## 2 Methods

This is a cross‐sectional, observational study. A web‐based survey regarding end‐of‐life decisions was conducted among Swedish intensivists. The study is reported according to the CHEcklist for Reporting Results of Internet E‐Surveys (CHERRIES) [11].

### 2.1 Study Setting and National Conditions

The healthcare system in Sweden is free of charge for all citizens under the age of 20, including outpatient clinics and hospital care. For adults, a nominal fee is charged per hospital night, regardless of the intensity or type of care provided. Intensive care, advanced treatment or diagnostics do not incur any additional costs for patients or their families [12]. Consent is required for all somatic care with a few exceptions. In clinical practice, a pragmatic approach is often employed, with presumed consent unless explicitly refused. Written consent forms are rarely used.

The Swedish guidelines of where critically ill children are best treated state that younger children, especially those under the age of three with an expected need for mechanical ventilation for more than 48 h, should be transferred to a dedicated PICU. This is also recommended for children who require continuous renal replacement therapy or suffer from multi‐organ failure. Older children, especially those older than 12 years, weighing 40 kg or more, can be treated at an ICU with mixed adults and children. An individual assessment is made for the age groups between 3 and 12 [13]. Children in the neonatal period (0–30 days) are primarily cared for in neonatal intensive care units (NICUs). However, there are many exceptions where children younger than 30 days are cared for in the PICU, primarily due to a need for surgery early in life.

### 2.2 Participants

Participating hospitals were identified through the Swedish Intensive Care Registry (SIR), which contains prospectively collected data registered by healthcare professionals during ICU admissions, including PICU but excluding NICU.

The ICUs in Sweden that treated more than 10 children for more than 48 h during 2023 were approached through the head of department with a request to distribute a link to the web‐based survey to their employed physicians. All nine selected clinics, four PICUs and five ICUs accepted to participate.

The link to the web survey was distributed via e‐mail to all physicians in the selected departments. No further selection of individual physicians with a paediatric or intensive care profile was made, as there is a large overlap in staff during on‐call hours and such a selection could easily exclude potentially important respondents. The rationale for this approach and not a more focused selection of participants was that the intensive care community in Sweden is small and ensuring the participants' anonymity was considered more important. The survey was open to anyone with the correct web link, but it was not advertised in any way other than through direct e‐mails to the participants. No cookies, IP address or log file analysis was done to prevent duplicate answers. No time frame was measured.

### 2.3 Questionnaire

A first version of the questionnaire was constructed by the first author (M.A.), based on themes from earlier research [9, 13, 14]. The questionnaire consisted of five modules: participant characteristics; facilitating professional structures; assessment of different values in decision‐making; theoretical questions on the ethics of withholding or withdrawing LST; and case‐based questions relevant to the clinical context.

Face validity was established by having three co‐authors (J.M., A.G.C. and L.B.) review the questionnaire. To confirm validity, a pilot study was conducted with six experienced intensivists who completed the questionnaire and were then asked to provide feedback on any questions that were unclear or difficult to answer. The pilot questionnaires were excluded from the results and the questionnaire was revised according to the feedback provided.

The questionnaire consisted of 33 items distributed on five pages (Supplementary 1). Answer options were diverse depending on the question. A five‐step Likert scale was used whenever appropriate; all questions had a ‘Rather not say’ option. There was no randomisation, alternation or adaptive questioning. It was possible to go back and change answers before submitting; no completeness checks were performed. The questionnaire was then transcribed into survey software, Esmaker (Entergate AB, Halmstad, Sweden).

The first page of the questionnaire was an informed consent form that provided information about the study and the time required to complete the questionnaire and clearly stated that participation was voluntary and without reimbursement. The purpose of the study and contact details of the investigator (M.A.) were provided. Information about anonymity was also provided in the consent form, but no information about data storage was provided.

### 2.4 Data Collection

The study was conducted from August 29 to September 22, 2024. Three weeks after the initial invitation, a reminder was sent out by e‐mail. The data was collected and stored anonymously on a password‐protected computer.

### 2.5 Data Analysis

The data was retrieved from Esmaker (Entergate AB, Halmstad, Sweden). Descriptive statistics were performed using Microsoft Excel (Microsoft 365 MSO version 2408) and RStudio (version 2025), an integrated development environment for R, developed by Posit Software (PBC, Boston, MA; URL: http://www.posit.co/).

### 2.6 Ethics and Registration

The study was approved by the Swedish Ethical Review Authority on August 14, 2024 (Ref# 2024‐01732‐01). The study, LSTPedSurvey, is registered on ClinicalTrials.gov as of 2024‐01‐16 under registration ID NCT06281743.

## 3 Results

### 3.1 Participants

The link to the web‐based questionnaire was distributed to 360 intensivists by e‐mail. In total, 55 complete answers were retrieved. Beyond this, five incomplete answers were detected and excluded from analysis. The participating physicians were experienced, with 62% of the respondents working as senior consultants (Table 1a).

**TABLE 1a:** Participant characteristics (*n* = 55).

Variable	*n*	%
Age		
< 35	2	4
36–50	23	42
51–65	28	51
> 65	2	4
Year of graduation		
Before 1999	22	40
2000–2010	31	56
After 2010	2	4
Country for medical degree (mark several options if appropriate)		
Sweden	43	78
Another EU country	12	22
Country outside of Europe	0	
Primarily working as		
On‐call Primary physician	18	33
Senior Consultant on‐call	34	62
Not on‐call	2	4
Don't want to answer	1	2
Clinical setting		
PICU (pediatric ICU)	30	55
ICU (mixed adult/child ICU)	24	44
Other	1	2
Completed full questionnaire	55	100

The participating hospitals were selected based on their case load the year before the survey (Table 1b). This selection of nine ICUs covered 87% of the children treated in ICU for more than 48 h and 74% of child deaths in ICUs in Sweden during the year 2023. The distribution of diagnoses in the respective ICUs is shown in Table 1c.

**TABLE 1b:** Number of recipients of the survey at each hospital, matched with the number of children treated in ICU 2023‐01‐01 to 2023‐12‐31.

	Recipients of the survey	Children in ICU total	Children > 48 h in ICU	Deaths in ICU
PICU				
C	48	539	237	9
E	35	525	208	9
F	12	348	132	6
H	20	389	140	8
ICU				
A	46	60	13	2
B	107	56	15	3
D	20	37	32	4
G	16	88	13	1
I	56	90	19	1
Sum	360	2132	809	43

*Note:* Swedish Intensive Care Registry, https://portal.icuregswe.org/utdata/2023.

**TABLE 1c:** Demographics for participating intensive care units 2023‐01‐01 to 2023‐12‐31.

	Congenital abnormalities and neonatal	Infections and resipratory	Musculoskeletal, metabolic and neurology	Trauma and intoxications	Circulatory	Oncology	GI and transplants	Symtomatic and unspecified
PICU								
C	101	177	87	40	55	24	9	44
E	246	91	47	28	50	23	20	19
F	244	34	14	11	22	18	5	2
H	23	124	50	54	27	14	16	83
ICU								
A	0	28	11	12	0	1	3	4
B	0	15	14	1	4	1	14	4
D	11	18	2	0	7	1	0	0
G	1	14	22	19	3	10	2	10
I	10	36	13	16	2	1	3	13
Sum	636	537	260	181	170	93	72	179

*Note:* Swedish Intensive Care Registry, https://portal.icuregswe.org/utdata/2023.

### 3.2 Attitudes

**FIGURE 1:**
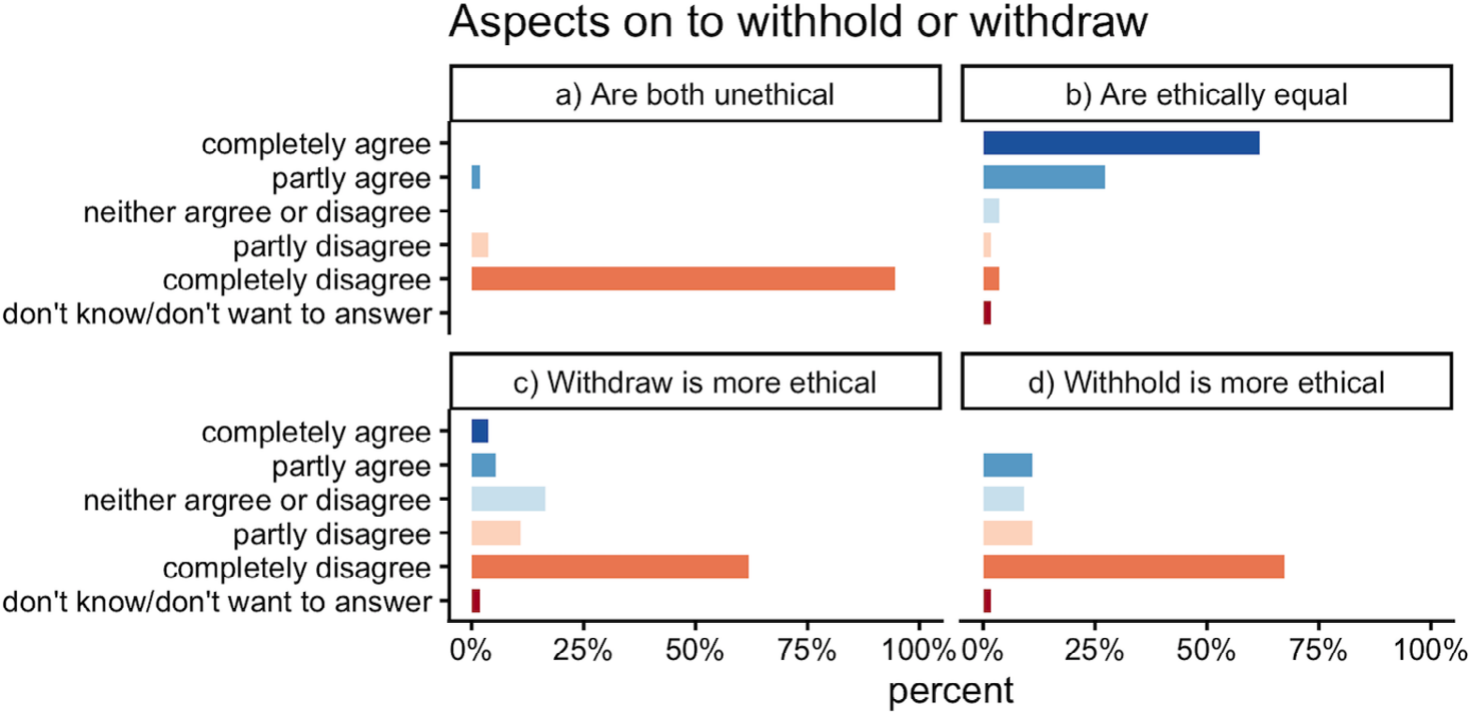
Regarding withholding or withdrawing life‐sustaining treatment. (a) To withhold OR withdraw life‐sustaining treatment is unethical. (b) To withhold and to withdraw life‐sustaining treatment are ethically equivalent. (c) To withdraw life‐sustaining treatment is more ethically acceptable than to withhold life‐sustaining treatment. (d) To withhold life‐sustaining treatment is more ethically acceptable than to withdraw life‐sustaining treatment. The exact percentage is given in Supplementary 1.

Ninety‐eight percent of respondents disagreed with the statement that both withholding and withdrawing LST should be considered unethical. Eighty‐nine percent agreed that there is no ethical difference between withholding and withdrawing. Regarding the statement that withholding is more ethical than withdrawing, 78% of respondents disagreed. Likewise, 73% disagreed that withdrawing is more ethical than withholding (Figure 1).

### 3.3 Values

In a theoretical context, when asked, ‘*What values are important for decisions to withhold or withdraw LST*?’, the child's wishes and what is in the child's best interest are considered the most important factors. The child's wishes are considered very important by 66% of respondents and important by 27%. The child's best interest is considered very important by 53% and important by 36%. The guardians' wishes are considered important by most respondents (66%) and very important by 16%.

**FIGURE 2:**
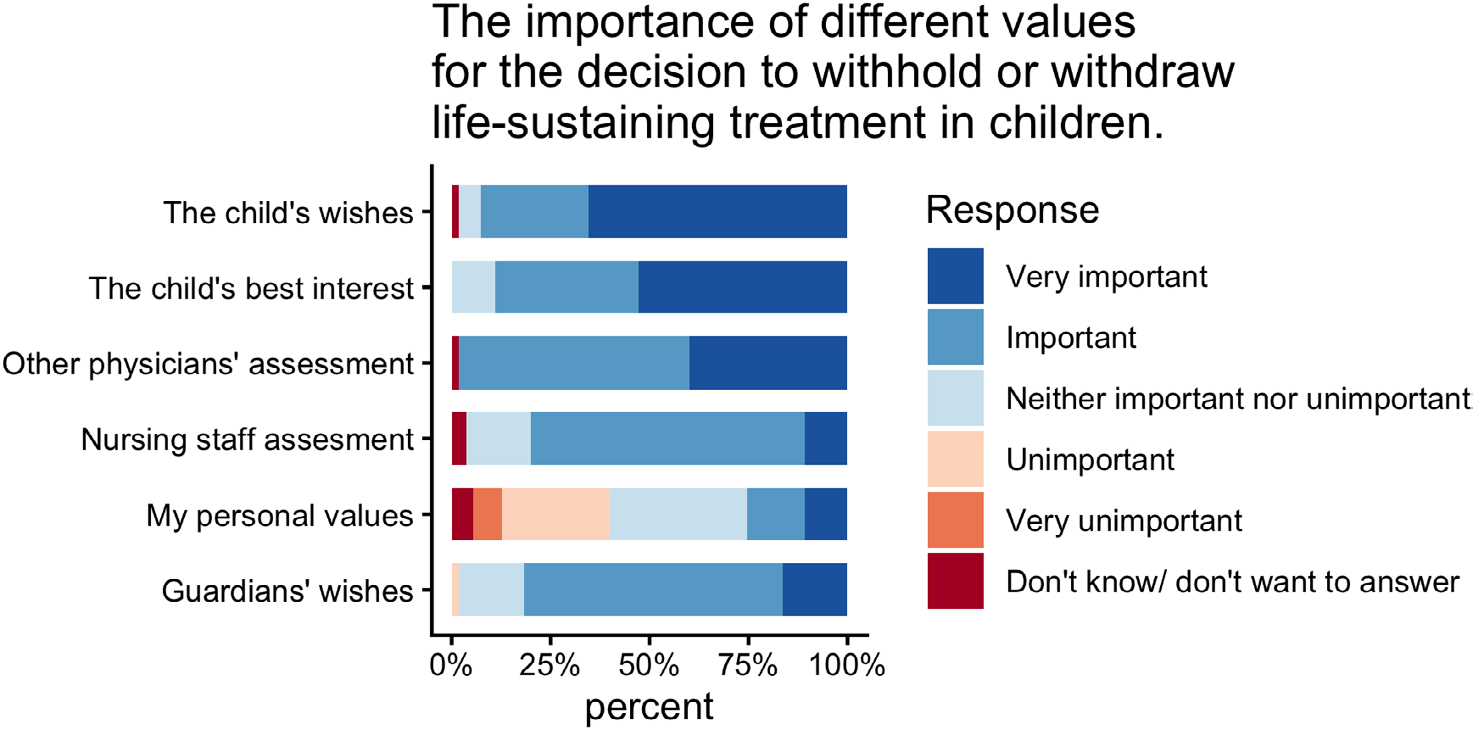
Regarding how important I consider different values to be when I take a stand in a decision to withhold or withdraw life‐sustaining treatment in children. The exact percentage is given in Supplementary 1.

Concerning professional stakeholders, the assessment of other physicians is considered very important to a greater extent than that of nursing staff (40% vs. 11%). Regarding the importance of the respondent's personal values, the answers were distributed as follows: important or very important (25%), unimportant or very unimportant (35%), undecided (35%) (Figure 2).

### 3.4 Attitudes Towards Conflict Scenarios

**FIGURE 3:**
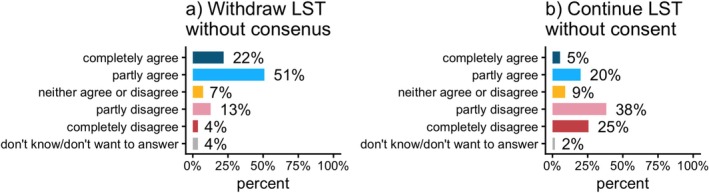
(a): ‘It is ethically acceptable to withdraw life‐sustaining treatment for a patient when both the patient and their guardians wish to continue treatment’. (b): ‘It is ethically acceptable to continue life‐sustaining treatment for a patient when both the patient and their guardians wish to withdraw it’.

We found that 73% of physicians considered it ethically acceptable to withdraw LST against the patients' and guardians' wishes, that is, withdraw without consensus (Figure 3a). Furthermore, we found that 25% of physicians considered it ethically acceptable to continue LST without the patients' and guardians' consent (Figure 3b).

### 3.5 Differences in Responses Between PICU and ICU Physicians

**FIGURE 4:**
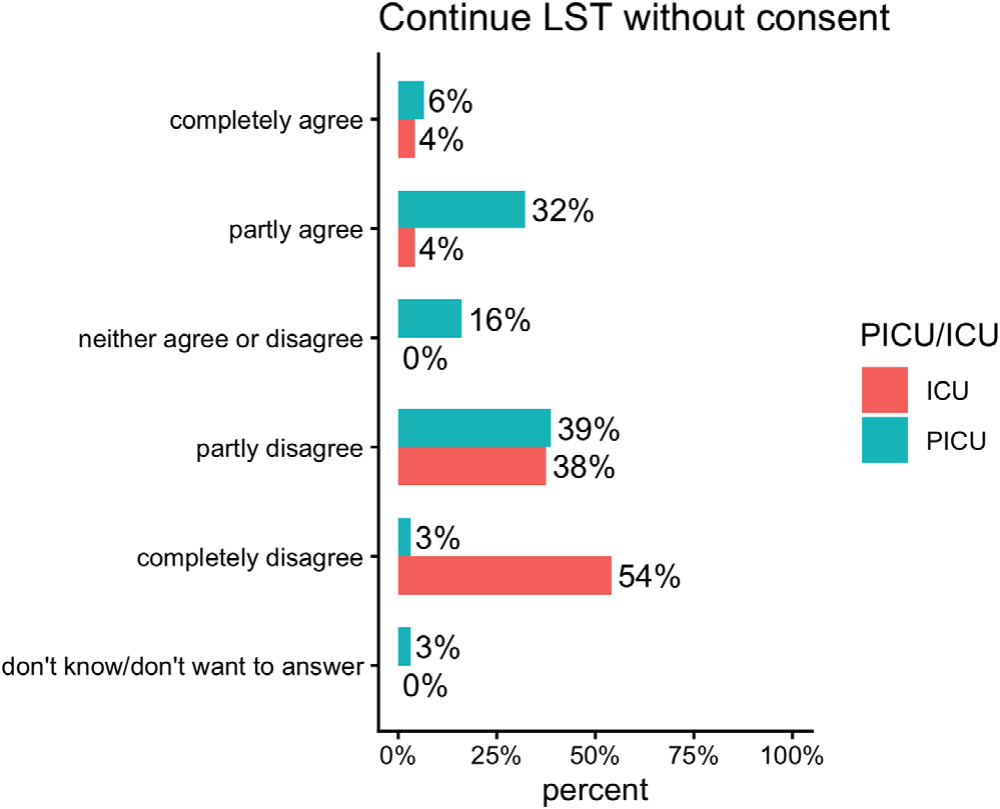
‘It is ethically acceptable to continue life‐sustaining treatment for a patient when both the patient and their guardians wish to withdraw it’. ICU = Intensive Care Unit with children and adult patients. PICU = designated Paediatric Intensive Care Unit.

There were differences in responses between physicians working in the PICU and those working in the ICU. Concerning the statement, ‘It is ethically acceptable to continue LST even though both the patient and guardians want to withdraw’, 92% of the ICU physicians disagreed. Among PICU physicians, 42% disagreed and 38% agreed, while 16% were undecided (Figure 4).

### 3.6 Experiences

**FIGURE 5:**
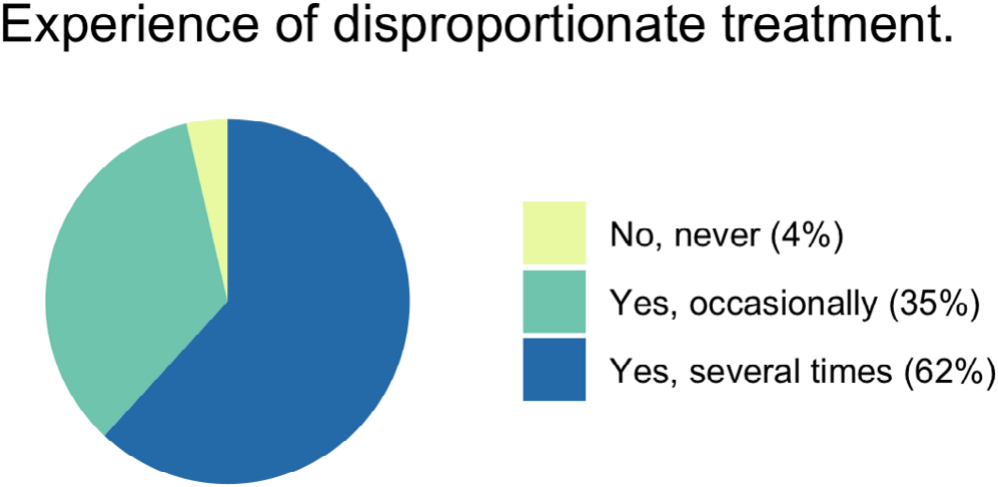
‘I have at least once treated a child where I have felt that the child is receiving disproportionate care in relation to their prognosis’.

**FIGURE 6:**
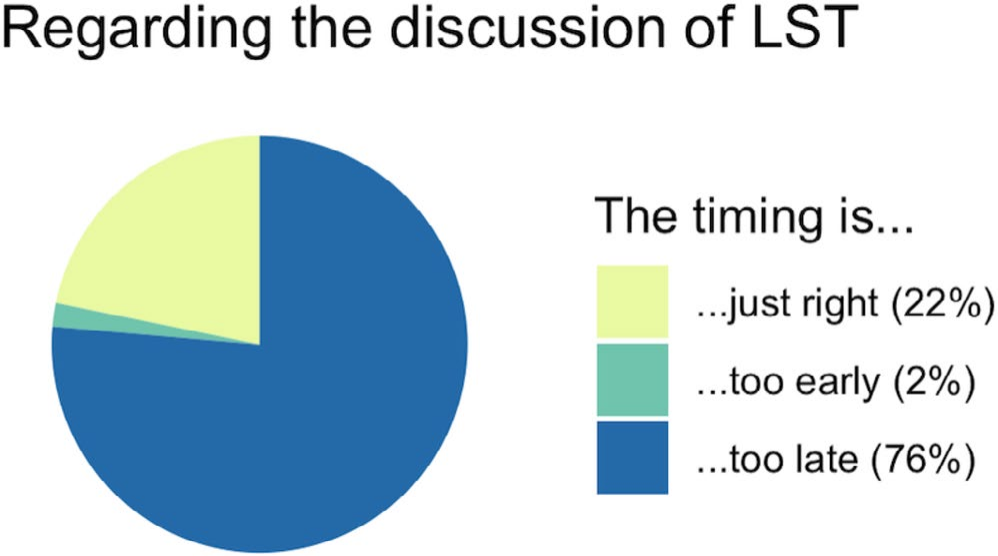
‘According to my experience, the timing for a discussion about a decision to withhold or withdraw life‐sustaining treatment is often…just right/too early/too late’.

Ninety‐three percent of respondents had personal experience with end‐of‐life decision‐making for children (Table 2). Ninety‐six percent of respondents had at least once treated a child whom they felt was receiving disproportionate treatment (Figure 5). Regarding the timing of discussions on end‐of‐life decision‐making, 76% of respondents reported that it is often too late (Figure 6).

**TABLE 2:** Participants experiences (*n* = 55).

	No, never		Yes, occasionally		Yes, several times	
	*n*	%	*n*	%	*n*	%
I have treated children < 15 years in ICU in the past 12 months.	0		3	6	52	95
I have been directly involved in the care of a child with a decision to withhold or withdraw life‐sustaining treatment.	1	2	11	20	43	78
I have at some point been directly involved in the decision‐making process to withhold or withdraw life‐sustaining treatment for a child.	4	7	12	22	39	71
I have been directly involved in a clinical case that has been revised by the Medical Responsibility Board (IVO in Swedish), regardless of which outcome the revision resulted in.	28	51	24	44	3	6
I have at least once cared for a child where I have felt that the child is receiving disproportionate care in relation to the prognosis.	2	4	19	35	34	62

## 4 Discussion

In this cross‐sectional observational study, we aimed to identify the attitudes and experiences of Swedish intensivists regarding the decision‐making process to withhold or withdraw LST in critically ill children.

In this study, there was broad agreement that withholding or withdrawing LST is considered ethically acceptable, though somewhat challenging. There was also a clear majority who believed that there is no ethically relevant difference between withdraw and withhold, which aligns with earlier studies in Europe [10]. In the questions comparing withholding and withdrawing separately, we found a slight variability in the answers, reflecting the complexity of the concept.

Despite there being no ethically relevant difference between withhold and withdraw, there might be a psychological difference between the loss of something compared to never gaining it. This complexity is previously known, as described in a recent study by Strand et al. [14].

There has been a shift from paternalistic decision‐making to a more patient‐centred view in all treatment decisions, both in paediatric and adult care in Europe during the past decade [15].

Concerning children, categorical non‐treatment strategies for specific patient groups were practised 50 years ago but have now been replaced by individual assessment [16].

A more patient‐centred decision‐making was evident in our study, as the child's own wishes were considered the most important value in the decision‐making. In the scope of this study, we have not investigated further the nuances involved in interpreting a child's wish. Age, maturity, cognitive capacity and the risk of coercion are different aspects that may influence the interpretation of a child's wish. Notably, the patient's best interests were considered more important than the guardians' wishes in the decision.

When these questions were combined into clinical scenarios, we found that if the case involved withdrawing LST against the patients' and guardians' wishes, a clear majority agreed that it is ethically acceptable to withdraw without consensus. This is supported by the Swedish Medical Services Act [6], which limits a patient's right to demand prolonged treatment if deemed futile.

When the case involved continuing LST despite the patient and guardians' wishes to withdraw, the responses were ambiguous. This situation is less common and more complicated from a judicial perspective. This is exemplified by a situation in which treatment is available and indicated, but the patient and parents refuse due to religious or other convictions. These situations are uncommon and complex, which may explain the wider variation in the answers. Regarding the same question to continue without consent, there was a difference in responses between the group working in designated PICUs and those working in ICUs. 92% of ICU physicians disagreed with continuing LST without consent. It could be speculated that the ICU physicians perceive the wishes of a minor patient and their guardians as equal to those of an autonomous adult. The PICU physicians had more diverse responses; almost 40% agreed that it is ethically acceptable to continue LST without the patient's and guardians' consent. The case did not state the child's age, which would probably have influenced the response. The ICU physicians will most likely associate the question with an older child than a PICU physician, due to the demographic differences in their respective wards. From this study, there is no data to determine whether differences in responses from PICU and ICU are due to different exposure or to differences in ethical stance. In the ICU, exposure to end‐of‐life decision‐making is more frequent than in the PICU. Whereas ethical issues concerning child autonomy and guardianship are more common in the PICU.

A previous study by Martins et al. [17] indicates that although an adolescent has equal capacity to both consent to and refuse treatment, it is argued that the assessment of their decision‐making capacity needs to be more robust in cases where their refusal of treatment might be detrimental to their health.

The experience of providing disproportionate care in relation to the patient's prognosis is widespread, according to our study and earlier studies in adult ICU settings [8]. Three out of four respondents believe that the timing for discussing end‐of‐life decisions is often too late, which aligns with earlier studies [10]. This suggests that disproportionately too much care is more common than too little in an ICU setting, which can be expected given the intensity of care provided in the ICU.

For individual patients and their families, a timely, transparent decision‐making process is important to avoid unnecessary and painful treatment. Furthermore, it provides families with the opportunity to make informed decisions about where and how to spend the child's remaining days. At an organisational level, ensuring that the right patient receives the proper treatment is important for efficient resource management.

## 5 Strengths and Limitations

The main limitation of this study lies in the response rate and that the potential bias induced by non‐responders is a source of uncertainty.

However, it could be argued that since the respondents were very experienced, physicians with clinical experience in the matter at hand and an interest in the subject have answered to a high degree. Nevertheless, caution must be applied in the interpretation and generalizability of the results.

In evaluating the validity of the response rate in a structured framework suggested by Holtom et al. [18], the main strength of the study is the participants' qualifications. Distribution through departments ensured that the survey reached a large part of the eligible respondents. To enhance the quality of participant motivation, a strategy is to keep the investigator–participant relationship anonymous and limit the number of reminders, thereby reducing the risk of coercive or forced responses. This is especially important given the complex judgement required to answer this type of questionnaire. In a national context, the number of respondents (55) can be related to the number of child ICU deaths per annum (60–80) in Sweden and to the number of members of the national society for paediatric anaesthesia and intensive care (135) at the time the survey was conducted.

## 6 Conclusion

We found that Swedish intensivists share common ground regarding attitudes towards end‐of‐life decision‐making in children. The most important factor for Swedish intensivists when making an end‐of‐life decision for a child is the patient's own wishes and what is considered to be in the child's best interest. There are differences in how stakeholders are considered in cases of continuing or discontinuing LST; in this specific regard, there are differences in attitudes between PICU and ICU physicians. The experience of disproportionate care and a late initiation of end‐of‐life discussions is common. We found that withholding and withdrawing LST appropriately is considered ethically acceptable, warranting further research into the details of this issue.

## Author Contributions

Conception and study design: M.A., J.M., A.G.C., L.B. Analysing: M.A., J.M., L.B. Writing first draft: M.A., L.B. Critical review and processing of manuscript and approval of final manuscript: All authors.

## Funding

This study was funded by grants from the following: The Swedish governmental funding of clinical research (ALF) ALFGBG‐1028656, by departmental funding, by Wilhelm and Martina Lundgren, Science Fund 2024‐SA‐4447, 2025‐SA‐4848.

## Conflicts of Interest

The authors declare no conflicts of interest.

## Supporting information


**Supplementary 1** Distribution of answers for the items of the questionnaire that are not reported in Table 1 or 2.

## Data Availability

The data that support the findings of this study are available on request from the corresponding author. The data are not publicly available due to privacy or ethical restrictions.

## References

[1] “Ethical Guidelines for Decisions on Withholding or Withdrawing Life‐Sustaining Treatment,” https://www.sls.se/media/apumorpm/sls_ssf_riktlinjer_avbryta_avsta_2018‐1.pdf.

[2] “Socialstyrelsen's Regulations and General Recommendations on Life‐Sustaining Treatment,” https://www.socialstyrelsen.se/publikationer/sosfs‐20117‐socialstyrelsens‐foreskrifter‐och‐allmanna‐rad‐om‐livsuppehallande‐behandling‐‐2011‐6‐26/.

[3] “Treatment Strategies for Life Support in Intensive Care,” https://sfai.se/wp‐content/uploads/files/22‐2%20Treatment_strategy.pdf.

[4] “Childrens Outdata Portal, Number of Admissions, Treatment Result Deceased,” https://portal.icuregswe.org/barn/sv/report/antalvtf.

[5] “Deaths by Age, Gender and Year,” https://www.statistikdatabasen.scb.se/pxweb/sv/ssd/START__BE__BE0101__BE0101I/DodaHandelseK/table/tableViewLayout1/.

[6] “Health and Medical Services Act (2017:30),” https://www.riksdagen.se/sv/dokument‐och‐lagar/dokument/svensk‐forfattningssamling/halso‐och‐sjukvardslag‐201730_sfs‐2017‐30/.

[7] A. Nordenskjöld Syrous , A. Ågård , M. Kock Redfors , S. Naredi , and L. Block , “Swedish Intensivists' Experiences and Attitudes Regarding End‐Of‐Life Decisions,” Acta Anaesthesiologica Scandinavica 64, no. 5 (2020): 656–662. 31954072 10.1111/aas.13549

[8] R. D. Piers , E. Azoulay , B. Ricou , et al., “Perceptions of Appropriateness of Care Among European and Israeli Intensive Care Unit Nurses and Physicians,” Journal of the American Medical Association 306, no. 24 (2011): 2694–2703. 22203538 10.1001/jama.2011.1888

[9] D. Benoit , H. I. Jensen , J. Malmgren , et al., “Outcome in Patients Perceived as Receiving Excessive Care Across Different Ethical Climates: A Prospective Study in 68 Intensive Care Units in Europe and the USA,” Intensive Care Medicine 44 (2018): 1039–1049. 29808345 10.1007/s00134-018-5231-8 PMC6061457

[10] A. Zanin , J. Brierley , J. M. Latour , and O. Gawronski , “End‐Of‐Life Decisions and Practices as Viewed by Health Professionals in Pediatric Critical Care: A European Survey Study,” Frontiers in Pediatrics [Internet] 10 (2023): 1–9. 10.3389/fped.2022.1067860 PMC9872024 36704131

[11] G. Eysenbach , “Improving the Quality of Web Surveys: The Checklist for Reporting Results of Internet E‐Surveys (CHERRIES),” Journal of Medical Internet Research 6, no. 3 (2004): e34. 15471760 10.2196/jmir.6.3.e34 PMC1550605

[12] “Patientavgifter‐I‐Vastra‐Gotaland,” https://www.1177.se/Vastra‐Gotaland/sa‐fungerar‐varden/kostnader‐och‐ersattningar/patientavgifter‐i‐vastra‐gotaland/#section‐4186.

[13] “Riktlinje‐angående‐organisation‐av‐barnintensivvård‐i‐Sverige,” https://sfai.se/wp‐content/uploads/2015/02/Riktlinje‐ang%C3%A5ende‐organisation‐av‐barnintensivv%C3%A5rd‐i‐Sverige.pdf.

[14] L. Strand , L. Sandman , E. Persson , D. Andersson , A. C. Nedlund , and G. Tinghög , “Withdrawing Versus Withholding Treatments in Medical Reimbursement Decisions: A Study on Public Attitudes,” Medical Decision Making 44, no. 6 (2024): 641–648. 38912645 10.1177/0272989X241258195 PMC11346081

[15] C. L. Sprung , R. D. Truog , J. R. Curtis , et al., “Seeking Worldwide Professional Consensus on the Principles of End‐of‐Life Care for the Critically Ill. The Consensus for Worldwide End‐of‐Life Practice for Patients in Intensive Care Units (WELPICUS) Study,” American Journal of Respiratory and Critical Care Medicine 190, no. 8 (2014): 855–866. 25162767 10.1164/rccm.201403-0593CC

[16] L. Gillam , “Fifty Years of Paediatric Ethics,” Journal of Paediatrics and Child Health 51, no. 1 (2015): 8–11. 25586839 10.1111/jpc.12793

[17] G. H. Martins , K. Eler , A. Albuquerque , and R. Nunes , “Is the Capacity to Consent Different From the Capacity to Refuse Treatments and Procedures in Adolescence?,” Jornal de Pediatria 101, no. 4 (2025): 501–510. 40378870 10.1016/j.jped.2025.04.004 PMC12276603

[18] B. Holtom , Y. Baruch , H. Aguinis , and G. A Ballinger , “Survey Response Rates: Trends and a Validity Assessment Framework,” Human Relations 75, no. 8 (2022): 1560–1584.

