# Supplementary material for: Swedish Intensive Care Physicians' Attitudes Towards Withholding or Withdrawing Life‐Sustaining Treatment in Critically Ill Children
Source: Acta Anaesthesiol Scand. 2026 May 15;70:e70256. doi: 10.1111/aas.70256 (PMC13178199; doi:10.1111/aas.70256)
Supplement: Supplementary file 1 — Supplementary 1 Distribution of answers for the items of the questionnaire that are not reported in Table 1 or 2. [file AAS-70-0-s001.pdf]

# Supplementary 1

Distribution of answers for the items of the questionnaire that are not reported in Table 1 or 2.

| n (%) n=55 | Very High | High | Neither high nor low | Low | Very low | Don't know/ Don't want to answer |
|---|---|---|---|---|---|---|
| I value my own competence in decision-making to withhold or withdraw life-sustaining treatment in children. | 9 (16%) | 22 (40%) | 19 (35%) | 2 (4%) | 3 (6%) | 0 |

| n (%) n=55 | Very confident | Confident | Neither confident nor insecure | Insecure | Very insecure | Don't know/ Don't want to answer |
|---|---|---|---|---|---|---|
| I feel confident in the task of making decisions to withhold or withdraw life-sustaining treatment in children. | 8 (15%) | 26 (47%) | 14 (26%) | 4 (7%) | 3 (6%) | 0 |
| I feel confident in making decisions to withhold or withdraw life-sustaining treatment from the following perspectives: | | | | | | |
| Morally (Is this right?) | 11 (20%) | 34 (62%) | 5 (9%) | 5 (9%) | 0 | 0 |
| Existentially (Do I have the right to?) | 9 (16%) | 29 (53%) | 13 (24%) | 3 (6%) | 1 (2%) | 0 |
| Medically (Is the prognosis right?) | 6 (11%) | 30 (55%) | 14 (26%) | 3 (6%) | 2 (4%) | 0 |
| Legally (Am I allowed to do like this?) | 11 (20%) | 31 (56%) | 10 (18%) | 2 (4%) | 1 (2%) | 0 |
| Emotionally (I am upset by having to take these decisions) | 10 (18%) | 27 (49%) | 13 (24%) | 2 (4%) | 2 (4%) | 1 (2%) |
| Influence (I am intrusted with these types of decisions from my closest colleagues) | 16 (29%) | 29 (53%) | 5 (9%) | 3 (6%) | 1 (2%) | 1 (2%) |

| n (%) n=55 | | | | | | |
|---|---|---|---|---|---|---|
| Regarding how important I consider different values are when I take a stand in a decision to withhold or withdraw life-sustaining treatment in children. | Very important | Important | Neither important nor unimportant | Unimportant | Very Unimportant | Don't know/ Don't want to answer |
| My own personal values | 6 (11%) | 8 (15%) | 19 (35%) | 15 (27%) | 4 (7%) | 3 (6%) |
| The other physicians' assessment | 22 (40%) | 32 (58%) | 0 | 0 | 0 | 1 (2%) |
| The nursing staffs' assessment | 6 (11%) | 38 (69%) | 9 (17%) | 0 | 0 | 2 (4%) |
| The guardians' wishes | 9 (16%) | 36 (66%) | 9 (16%) | 1 (2%) | 0 | 0 |
| The child's own wish | 36 (66%) | 15 (27%) | 3 (6%) | 0 | 0 | 1 (2%) |
| What I believe to be in the child's best interest | 29 (53%) | 20 (36%) | 6 (11%) | 0 | 0 | 0 |
| The child's expected survival, even with intensive care | 39 (71%) | 15 (27%) | 1 (2%) | 0 | 0 | 0 |
| The child's expected quality of life if surviving ICU | 41 (75%) | 14 (26%) | 0 | 0 | 0 | 0 |
| The child's expected neurological outcome if surviving ICU | 28 (51%) | 26 (47%) | 1 (2%) | 0 | 0 | 0 |

| n (%) n=55 | Strongly agree | Agree | Neither agree nor disagree | Disagree | Strongly disagree | Don't know/ Don't want to answer |
|---|---|---|---|---|---|---|
| I feel that I have received sufficient in-service training on how decisions to withhold or withdraw life-sustaining treatment are made. | 9 (16%) | 25 (46%) | 10 (18%) | 5 (9%) | 6 (11%) | 0 |
| I feel that I receive sufficient support to be able to initiate discussions to withhold or withdraw life-sustaining treatment… | | | | | | |
| …from my employer | 28 (51%) | 16 (29%) | 0 | 5 (9%) | 5 (9%) | 0 |
| …from my physician colleagues in intensive care | 38 (69%) | 15 (27%) | 0 | 1 (2%) | 1 (2%) | 0 |
| …from my physician colleagues from the patient's referring wards | 8 (15%) | 29 (53%) | 0 | 12 (21%) | 6 (11%) | 0 |
| It is common for the nursing staff to question… | | | | | | |
| …why intensive care is continued | 22 (40%) | 27 (49%) | 4 (7%) | 1 (2%) | 1 (2%) | 0 |
| …why there are limitations to the intensive care | 2 (4%) | 15 (27%) | 9 (17%) | 17 (31%) | 12 (22%) | 0 |
| It is uncommon for the nursing staff to question the strategy of the intensive care in any way | 5 (9%) | 13 (24%) | 9 (16%) | 28 (51%) | 0 | 0 |

| n (%) n=55 | | | | | | |
|---|---|---|---|---|---|---|
| There is broad agreement that medico-ethical decisions should focus on the patient's best interest. But it isn't always that the different stakeholders agree on what is in the patient's best interest. The physicians are always responsible for a decision to withhold or withdraw life-sustaining treatment. It is ethically acceptable to… | Strongly agree | Agree | Neither agree or disagree | Disagree | Strongly disagree | Don't know/ Don't want to answer |
| …withdraw life-sustaining treatment even though the patient and guardians want to continue treatment. | 12 (22%) | 28 (51%) | 4 (7%) | 7 (13%) | 2 (4%) | 2 (4%) |
| …continue life-sustaining treatment for a patient where both patient and guardians want to withdraw. | 3 (6%) | 11 (20%) | 5 (9%) | 21 (38%) | 14 (26%) | 1 (2%) |
| …continue life-sustaining treatment in a situation where I don't think it benefits the patient, but the guardians oppose to withdraw treatment, and the patients wishes are unknown. | 1 (2%) | 18 (33%) | 10 (18%) | 16 (29%) | 10 (18%) | 0 |
| …withdraw life-sustaining treatment because the patient wishes it, for a 14-year-old patient who is considered autonomous, despite the guardians opposing to withdraw treatment. | 6 (11%) | 25 (46%) | 7 (13%) | 11 (20%) | 5 (9%) | 1 (2%) |

| n (%) n=55 | | | | | | |
|---|---|---|---|---|---|---|
| Conscientious objection means that health care staff have the right to renounce certain tasks that ordinarily come with a position due to religious or ideological beliefs. In Swedish health care we do not have conscientious objection. | Strongly agree | Agree | Neither agree or disagree | Disagree | Strongly disagree | Don't know/ Don't want to answer |
| I am positive to that we don't have conscientious objection in Swedish healthcare. | 41 (75%) | 10 (18%) | 1 (2%) | 3 (6%) | 0 | 0 |

| n (%) n=55 | | | | | | |
|---|---|---|---|---|---|---|
| Regarding to withhold or withdraw life-sustaining treatment. | Strongly agree | Agree | Neither agree or disagree | Disagree | Strongly disagree | Don't know/ Don't want to answer |
| To withhold and to withdraw life-sustaining treatment are ethically equivalent. | 34 (62%) | 15 (27%) | 2 (4%) | 1 (2%) | 2 (4%) | 1 (2%) |
| To withhold OR withdraw life-sustaining treatment is unethical. | 0 | 1 (2%) | 0 | 2 (4%) | 52 (95%) | 0 |
| To withhold life-sustaining treatment is more ethically acceptable than to withdraw life-sustaining treatment. | 0 | 6 (11%) | 5 (9%) | 6 (11%) | 37 (67%) | 1 (2%) |
| To withdraw life-sustaining treatment is more ethically acceptable than to withhold life-sustaining treatment. | 2 (4%) | 3 (6%) | 9 (16%) | 6 (11%) | 34 (62%) | 1 (2%) |

| n (%) n=55 | | | | | | |
|---|---|---|---|---|---|---|
| | Strongly agree | Agree | Neither agree or disagree | Disagree | Strongly disagree | Don't know/ Don't want to answer |
| Concerning cardiac arrest where there might be a reversible iatrogenic cause to the cardiac arrest one should always do cardio-pulmonary-resuscitation regardless of if the patient has a treatment limitation decision to withhold cardio-pulmonary-resuscitation. | 2 (4%) | 11 (20%) | 7 13%) | 19 (35%) | 15 (27%) | 1 (2%) |

| n (%) n=55 | | | | | | |
|---|---|---|---|---|---|---|
| Concerning consensus in a discussion to withhold or withdraw life-sustaining treatment. In my experience… | Strongly agree | Agree | Neither agree or disagree | Disagree | Strongly disagree | Don't know/ Don't want to answer |
| …the team is usually already in consensus when the issue is raised. | 8 (15%) | 24 (44%) | 8 (15%) | 12 (22%) | 2 (4%) | 1 (2%) |
| …the discussion is usually structured, constructive and quickly reaches consensus in the team. | 4 (7%) | 22 (40%) | 8 (15%) | 19 (35%) | 1 (2%) | 1 (2%) |
| …the discussion is emotionally charged, difficult and it takes a long time before the team reaches consensus. | 3 (6%) | 20 (36%) | 6 (11%) | 16 (29%) | 9 (16%) | 1 (2%) |

| Regarding the timing for a discussion to withhold or withdraw life-sustaining treatment. In my experience the timing for a discussion to withhold or withdraw life-sustaining treatment for a child is often… | n (%) n=55 |
| --- | --- |
| …too early. | 1 (2%) |
| …just right. | 12 (22%) |
| …too late. | 42 (76%) |

| Is there in the hospital where you work any supportive function where clinicians can turn with a patient-related ethical dilemma or issue and recieve help with an ethical analysis of the problem and suggested course of action, from those who are not themselves invested in the clinical case? | n (%) n=55 |
| --- | --- |
| No | (29%) |
| Yes, but I have never used it | (38%) |
| Yes, I have recieved support from it | (26%) |
| Don't know/Don't want to answer | (7%) |
